# Large-scale evaluation of cytochrome P450 2C9 mediated drug interaction potential with machine learning-based consensus modeling

**DOI:** 10.1007/s10822-020-00308-y

**Published:** 2020-03-27

**Authors:** Anita Rácz, György M. Keserű

**Affiliations:** 1 Plasma Chemistry Research Group, Research Centre for Natural Sciences, Magyar tudósok krt. 2, Budapest, 1117 Hungary; 2 Medicinal Chemistry Research Group, Research Centre for Natural Sciences, Magyar tudósok krt. 2., Budapest, 1117 Hungary

**Keywords:** ADME-tox, Cytochrome P450, CYP 2C9, Machine learning, Classification

## Abstract

Cytochrome P450 (CYP) enzymes play an important role in the metabolism of xenobiotics. Since they are connected to drug interactions, screening for potential inhibitors is of utmost importance in drug discovery settings. Our study provides an extensive classification model for P450-drug interactions with one of the most prominent members, the 2C9 isoenzyme. Our model involved the largest set of 45,000 molecules ever used for developing prediction models. The models are based on three different types of descriptors, (a) typical one, two and three dimensional molecular descriptors, (b) chemical and pharmacophore fingerprints and (c) interaction fingerprints with docking scores. Two machine learning algorithms, the boosted tree and the multilayer feedforward of resilient backpropagation network were used and compared based on their performances. The models were validated both internally and using external validation sets. The results showed that the consensus voting technique with custom probability thresholds could provide promising results even in large-scale cases without any restrictions on the applicability domain. Our best model was capable to predict the 2C9 inhibitory activity with the area under the receiver operating characteristic curve (AUC) of 0.85 and 0.84 for the internal and the external test sets, respectively. The chemical space covered with the largest available dataset has reached its limit encompassing publicly available bioactivity data for the 2C9 isoenzyme.

**Electronic supplementary material:**

The online version of this article (10.1007/s10822-020-00308-y) contains supplementary material, which is available to authorized users.

## Introduction

The cytochrome P450 (CYP) enzyme family plays an important role in the biotransformation of xenobiotics. CYPs are involved in the bulk of phase I metabolism processes. Moreover, CYPs are responsible for most of the drug–drug interactions, which can affect the efficacy and safety of drugs. Many drugs have been withdrawn from the market due to the inhibition of these enzymes [1, 2]. Until now, 57 CYP genes are identified in humans, in which the CYP1, 2, and 3 families have the biggest contribution in the metabolism of the foreign substances [3]. More than 95% of FDA-approved drugs are metabolized by six isoforms of these subfamilies [4, 5]. The CYP 2C subfamily is one of the most important CYP families, consisting of two main isoforms, 2C9 and 2C19. Specifically, 2C9 is connected to the hepatic clearance of 12–16% of the clinically relevant drugs [6]. Anti-inflammatory agents, such as diclofenac, ibuprofen and anticoagulant molecules such as progesterone are amongst the substrates of CYP 2C9 isoenzyme [7]. CYP 2C9 is preliminarily expressed in the liver and the small mucosa, and the amount of CYP 2C9 is 15–20% compared to the total amount of the expressed CYP enzymes [8].

In the past 20 years the number of in silico models for different metabolic endpoints on CYP enzymes have increased and made the drug discovery processes faster and more efficient. Out of these, quantitative structure activity relationship (QSAR) models represent the correlation of biological activity on the specific CYP isoenzyme of the molecules with different molecular representations (descriptors) [7]. In the case of the CYP 2C9 isoform, the range of the models starts with a CoMFA model and a relatively small dataset containing 26 molecules in total by Jones and coworkers from 1996 [9]. In the past decade the number and size of available datasets increased rapidly. However, the number of compounds involved in model development has stopped around 15,000. Usually, the time consuming 3D models (CoMFA, CoMSIA) operated with a much lower number of compounds [10]. In contrast, prediction models based on bigger databases have mostly integrated machine learning (deep learning) algorithms and their combinations. The calculated models for CYP 2C9 are connected either to (a) usually smaller in-house datasets/literature data [11, 12] or (b) freely available large databases (such as PubChem) [13]. Machine learning algorithms have highly increased the performance of predictive models compared to earlier studies and parallel with the covered chemical space, their applicability domain could be widely extended. The trends in the modeling phase are the use of support vector machines (SVM) [14, 15], the Naïve Bayes algorithm [16], neural networks [17] or tree-based algorithms such as a simple correlation and regression tree (CART) or a more complex random forest (RF) or boosted tree (BT) [18]. Moreover, combined models are also represented in the literature, where either the applied datasets [13] or the algorithms [17] are combined. A great selection of the previous models (not just for the 2C9 isoform) can be found in the recent review by Kato [4].

In our study, we have taken a next step to overcome the limitations of the covered chemical space and built a large-scale model with over 45,000 individual molecules, which combines all the available Pubchem databases for the 2C9 isoform. Moreover, we purposefully did not apply any applicability domain concept in the modeling phase (to avoid any limitation regarding the modelled chemical space), and combined several models into a consensus optimum based on different molecular descriptor sets and fingerprint types. Our primary aim was not just to build the largest available model for CYP 2C9, but also to showcase the advantages of probability threshold optimization and consensus modeling. Based on the results obtained we suggest that consensus voting technique with custom probability thresholds provides a robust and predictive model for discriminating compounds with high 2C9 related drug interaction potential.

## Materials and methods

### Data handling

The study is based on the publicly accessible CYP 2C9 bioactivity databases available in PubChem (NCBI), with the following AID numbers: 777, 1851 and 883 [19–21]. These datasets include the class memberships of the molecules as active/inactive depending on the ability of test compounds to inhibit the 2C9 conversion of the substrate luciferin-H to luciferin.

Data handling was started with the elimination of those molecules, which had no SMILES code, or their class memberships were inconclusive (indicated in the database). Moreover, duplicates (either with different or identical class memberships) were also excluded from the dataset. The dominant protonation state at a pH of 7.4 was assigned to each compound with ChemAxon Calculator (cxcalc) [22] and Scrödinger (LigPrep) [23]. Scrödinger (LigPrep) was used to generate the 3D structures of the molecules in the exact protonation states.

The largest dataset, AID 777 was used for model training and internal validation. Thus, the model was optimized for this dataset. The numbers of active and inactive molecules were balanced in the dataset; in this way the two-class classification model could be more robust and less biased [24]. All active molecules were included. Having a balanced dataset, the same number of inactives were selected by the Diversity Picker node (RDKit extension) in KNIME based on the MaxMin algorithm [25]. Finally, still more than 35,000 different molecules with appropriate 3D structures were included in model building.

### Molecular descriptors sets

Different descriptor sets were calculated for the datasets including (a) classical 1, 2 and 3D molecular descriptors and extended connectivity fingerprints (ECFP) with the use of DRAGON 7.0 [26], (b) interaction fingerprints and docking scores by Schrödinger software (Glide) [27, 28] and (c) pharmacophore fingerprints with ChemAxon’s fingerprint generator.

Classical molecular descriptors were filtered based on the inter-correlations with the use of 0.997 as the correlation limit [29], constant descriptors were also omitted. In total, 3740 standard molecular descriptors were included for modeling. The definition of the used molecular descriptors can be found in details in reference [30]. In case of the ECFP fingerprint, standard parameters were used in DRAGON 7.0, with a maximum radius of 4 and a fingerprint length of 1024 [31]. The PDB structure 5K7K [32] was prepared using the Protein Preparation Wizard of Schrödinger [33] briefly bond orders and hydrogens were assigned, missing loops and sidechains were modeled, protonation states at a pH of 7.4 were generated, H-bond geometries were optimized and a restrained minimization of the structures was carried out. Ligand docking was carried out with Glide SP (standard precision) for the whole dataset into the selected CYP 2C9 enzyme structure from PDB (Protein Data Bank) database (ID: 5K7K [32]), containing a potent inhibitor (with an IC_50_ value of 36 nM). One binding pose was kept for each molecule. The 3D structures of the ligands for the docking were prepared with Schrödinger (LigPrep) as mentioned above. The predicted binding poses were used for the calculation of interaction fingerprints Schrödinger Maestro [34]. Non-interacting residues and non-occurring interactions were filtered out from the interaction fingerprint vectors, which finally contained 176 bit positions [35]. Pharmacophore fingerprints were generated with the fingerprint generator module of ChemAxon.

The descriptors were grouped together in three datasets, containing: (a) the interaction fingerprint variables, together with the docking score (177 variables), (b) the classical molecular descriptors calculated with DRAGON 7.0 (3740 variables) and (c) the ECFP fingerprints together with the pharmacophore fingerprints (1234 variables).

### Machine learning algorithms and evaluation tools

We have selected two well-known and frequently used machine learning algorithms, the tree-based gradient boosted tree (BT) [36], and the neural network-based multi-layer feed-forward of resilient backpropagation network (RPropMLP) [37] for modeling. Both algorithms are included in the KNIME analytics platform [38].

The resilient backpropagation is an improved backpropagation algorithm, which is an essential part of multi-layered feed-forward networks. Backpropagation learning means a repetitive application of the chain rule in order to calculate the influence of each weight in the network, taking into account an arbitrary error function [37]. RPropMLP algorithm implements a local adaptation of the weight updates, in agreement to the behavior of the error function. As in all standard neural network models, the number of hidden layers and the number of hidden neurons per layer can be (and are worth to be) optimized. These two parameters were optimized for every training model in a loop cycle in KNIME to find their best combination. RPropMLP is in the following text to MLP for simplicity.

Gradient boosted tree (GBT) is a well-known and useful technique amongst the tree-based algorithms. The basic idea of GBT is the ensemble of decision trees, which are created for the prediction of the target vector. The main difference of the algorithm compared to the previous versions is that the weak learners (decision trees) are identified by gradients in the loss function instead of using high weights (as it is happening for example in the standard adaptive boosting algorithm). Gradient boosted tree was optimized with the tree depth (limitation of the tree levels) for the training model in a loop cycle with KNIME (parameter optimization loop with brute force) in the same way as for MLP. The best value of tree depth was determined for each case.

## Results and discussion

**Fig. 1:**
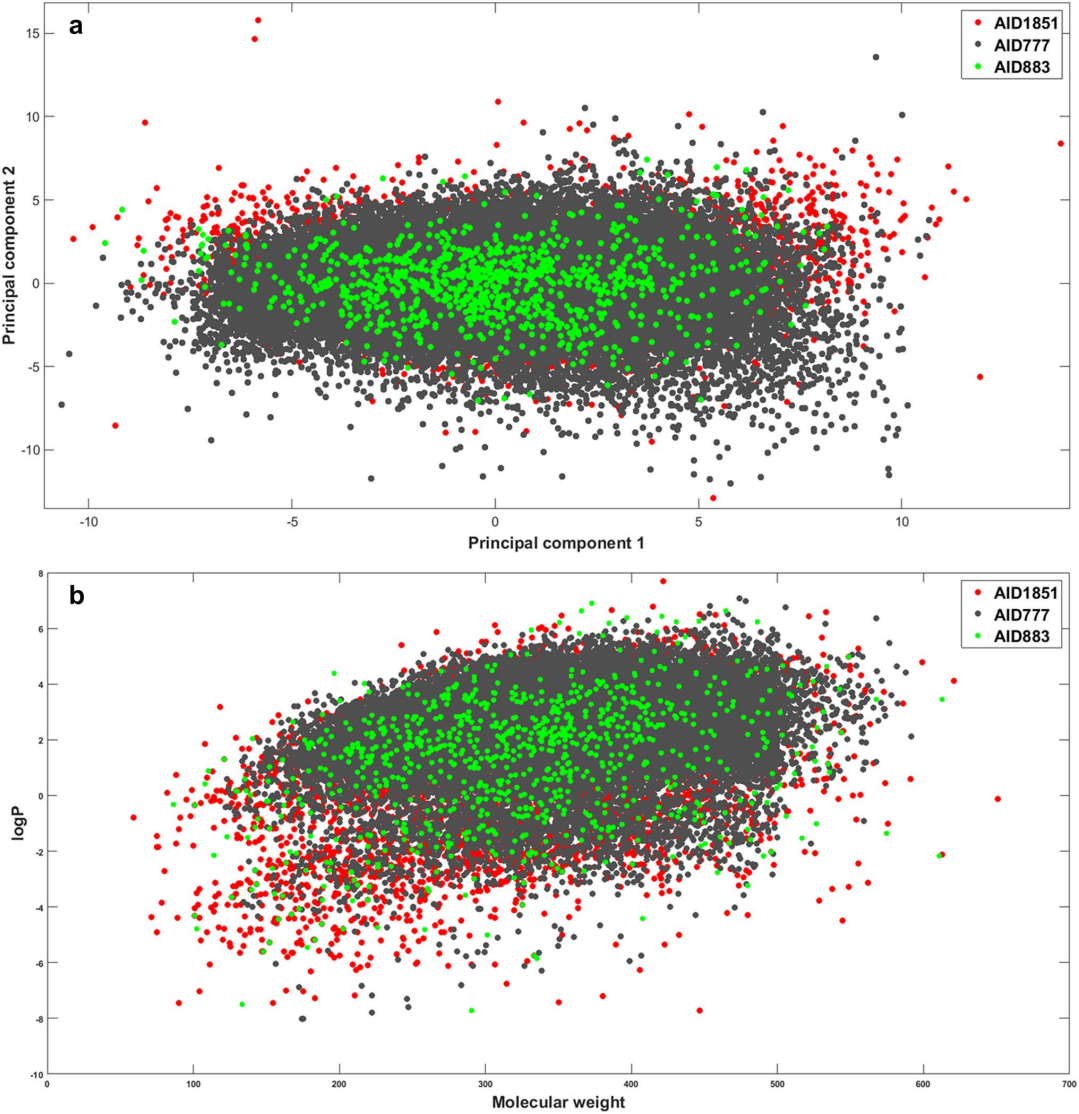
**a** Principal component analysis of the three PubChem datasets (grey: AID 777, red: AID 1851, green: AID 883). The first two principal component scores are plotted against each other. **b** Comparison of the three datasets based on the log P and molecular weight values of the molecules. The coloring is the same as in the previous case

First, the chemical space covered by the three applied datasets was visualized and compared. The evaluation was based on standard molecular descriptors (constitutional descriptors and molecular properties blocks in Dragon), and only unique molecules were included in each dataset. Principal component analysis was carried out and the first two principal component scores (PC) were plotted on a scatterplot (Fig. 1). Moreover the covered chemical space was also visualized in a scatterplot of logP against molecular weight values. The additional evaluation of drug-like properties can be seen in Supplementary material Figure S1. We can conclude that the chemical space coverage of AID 777 is much greater than the other two sets, thus AID 1851 and AID 883 were merged and applied together as an external test set. It can also be seen that the other two datasets occupy a subspace inside the AID 777, thus validating their use as the external set. In Fig. 1b it is shown that the AID777 dataset covers the largest area in the MW-logP space with much better sampling than that provided by AID1851 and AID883 sets.

Molecular descriptor variables were standardized for both of the algorithms. Five-fold stratified cross-validation (class ratios remains the same in each iteration), and internal validation with a 70% train–30% test ratio were used on the primary dataset (AID777). External validation was carried out with the merged set consisting of AID1851 and AID883. (The same data curation steps were applied to the external set, as outlined earlier.)

**Fig. 2:**
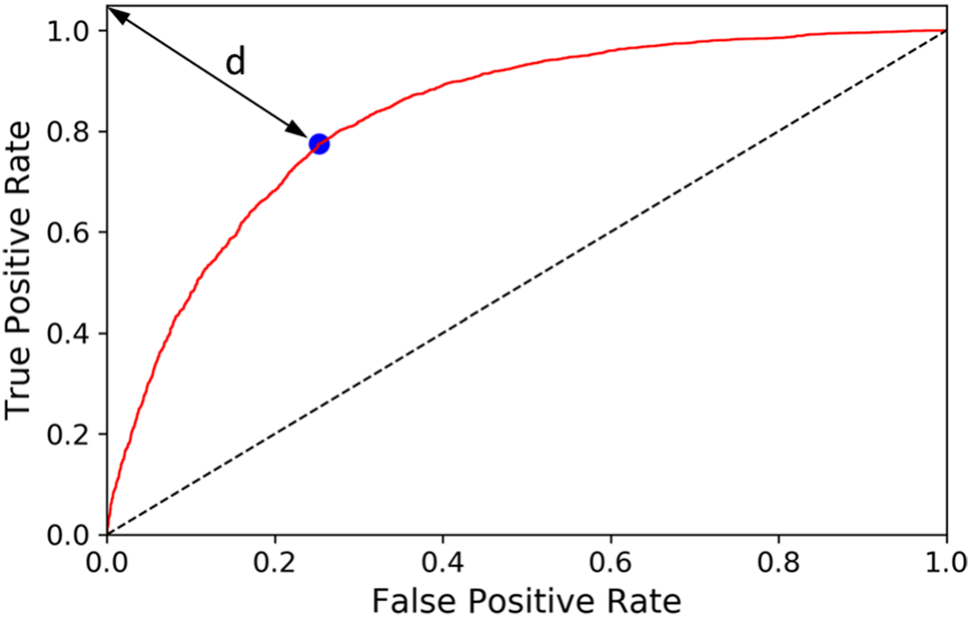
An example of the ROC curves with the threshold optimum determination (blue circle: point with the optimal probability value; d: distance between the optimal point and perfect classification)

The primary predicted class memberships were based on the individual class probabilities, with a threshold of 0.5. However, the optimum value of this threshold should be determined for each case. Models can perform much better if the best possible threshold is used, which can be actually higher or lower than 0.5. Thus, the probability threshold was determined based on the calculated receiver operating curves (ROC), defining the optimum value as the threshold value corresponding to point on the ROC curve with the minimum Euclidean distance (d) to the upper left corner of the plot (corresponding to perfect classification), see Fig. 2. Hence, the original class memberships were recalculated with the determined new thresholds for each dataset.

After the calculation of the primary classification models, consensus modeling (*consensus *1) was carried out based on the probability values for the active class, provided by each of the primary models. Minimum, maximum and average values were calculated for each molecule, and finally, ROC curves were plotted based on these new probability values.

Another version of consensus modeling was also applied (*consensus *2), where the molecules with inconclusive class memberships in the different models were excluded from the consensus model, keeping only those molecules where the predicted class memberships were the same for each primary model (after the threshold optimization). Minimum, maximum and average probability values for the active class were calculated for each molecule and ROC curves were plotted in the same way as for consensus 1 models.

**Fig. 3:**
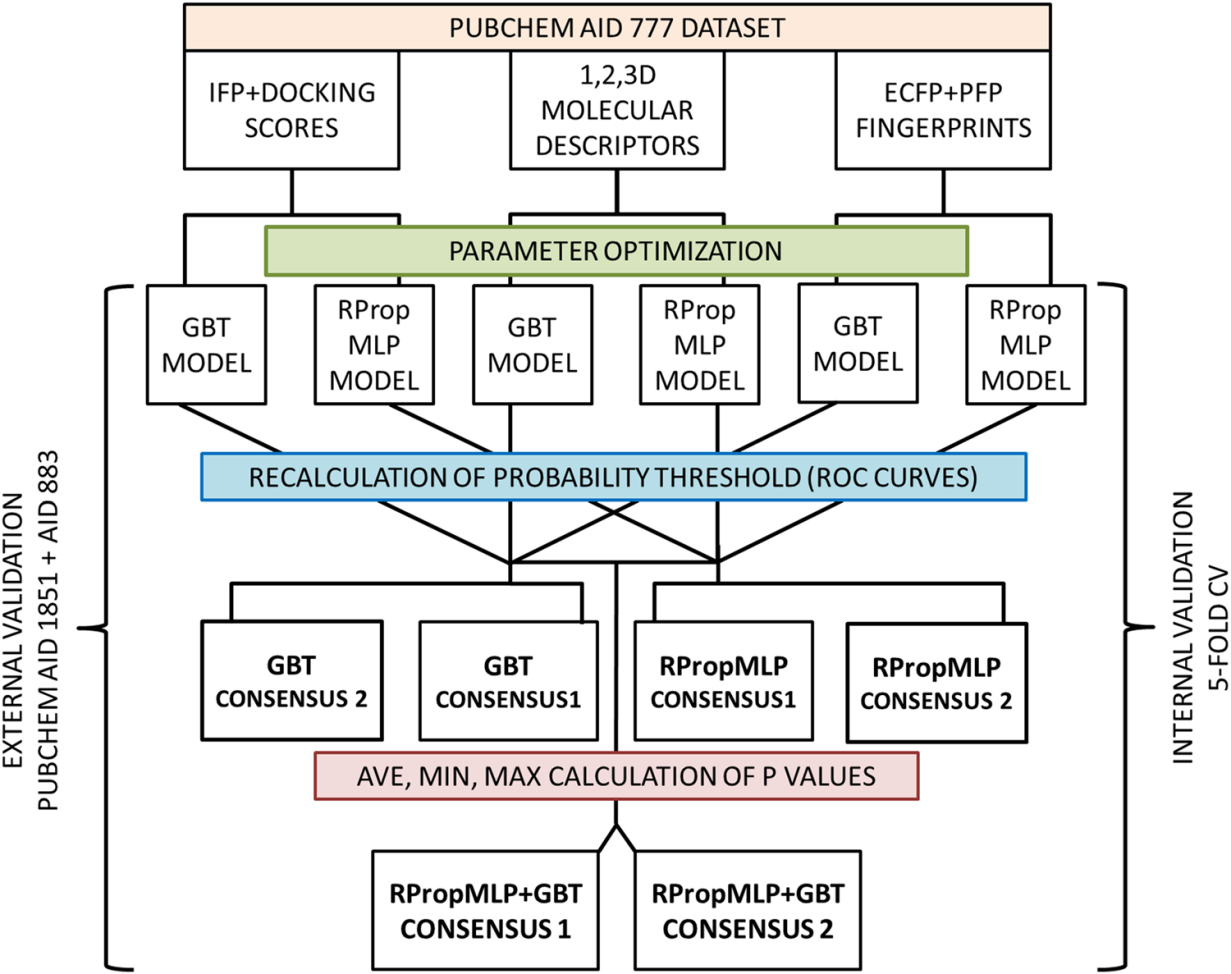
The complete modeling workflow. The explanation of the abbreviations can be found in the beginning of the manuscript

The complete workflow of the modeling is included in Fig. 3.

After data handling, 35,733 molecules were included in the models, with a ratio of actives to inactives of 46/54. The small difference from the 50–50 ratio was caused by the exclusion of those molecules, which failed during ligand docking. Thus, modeling was performed on each of the three different descriptor sets, with the training set containing 25,013 molecules, and the internal test set containing 10,720 molecules.

Class memberships were recalculated based on the determination of probability thresholds with the ROC curves of the original models. As a global performance metric, area under the ROC curve (AUC) was calculated for each model with the scikit-learn Python package [39].

The consensus models from the three primary datasets were generated with the calculation of the minimum, maximum and average values of the active probability values for each molecule. The performances of the primary models for the three descriptor sets and the calculated consensus 1 and consensus 2 models can be found in Table 1.

**Table 1:** AUC values of the prepared primary and consensus models for training, CV, internal and external validation

Method	Validation	Primary models			Consensus 1			Consensus 2		
		IFP + DS	ECFP + PFP	1D, 2D, 3D MD	Min	Ave	Max	Min	Ave	Max
MLP	Training	0.69	0.89	0.82	0.86	0.88	0.87	0.89	0.91	0.90
	CV	0.65	0.75	0.76	0.75	0.78	0.76	0.81	0.83	0.81
	Test	0.66	0.76	0.76	0.76	0.77	0.76	0.81	0.82	0.81
	External	0.65	0.74	0.74	0.75	0.76	0.74	0.79	0.81	0.80
GBT	Training	0.73	0.84	0.87	0.83	**0.85**	0.84	0.88	**0.89**	0.88
	CV	0.69	0.79	0.80	0.78	**0.79**	0.78	0.82	**0.84**	0.83
	Test	0.69	0.79	0.80	0.77	**0.79**	0.78	0.82	**0.83**	0.82
	External	0.67	0.75	0.76	0.75	**0.76**	0.73	0.80	**0.81**	0.80

Abbreviations can be found in the beginning of the text. The best models (based on the AUC of the validation sets) are marked with bold

In the comparison of the primary models, the ECFP + PFP and 1, 2, 3D molecular descriptors clearly outperformed the IFP + DS (docking score) versions. The average of the probability values was the best consensus option, not just for the consensus 1 models, but for the consensus 2 models as well. Gradient boosted tree performed slightly better for the primary models, in the consensus modeling part, the performances were very similar for the two algorithms. The validation part was successful; models performed excellently even for the external sets (with a relatively minor performance drop compared to training). The amount of molecules for each validation part, and the ratio of actives and excluded molecules for consensus 2 can be seen in Table 2. The number of molecules was the same as for the primary models, in the case of the consensus 1 models.

**Table 2:** The number of molecules in each validation part, and the number of active molecules in consensus 2 models

Samples	Validation	Consensus 1	Consensus 2	Active molecules in consensus 2 (%)	Ratio of excluded molecules (consensus 2)
MLP	Training	25,013	15,567	52.5	0.41
	CV	25,013	14,696	48.4	0.41
	Test	10,720	6608	49.6	0.38
	External	9983	5919	21.0	0.41
GBT	Training	25,013	17,348	51.8	0.31
	CV	25,013	16,587	48.1	0.34
	Test	10,720	7260	48.9	0.32
	External	9983	6013	22.1	0.40

The ratio of excluded molecules is assigned to consensus 2 models

In the final model building step, the consensus models of all the six primary models were calculated based on the active probability values and the assigned class memberships. Minimum, maximum and average of the probability values were compared in the consensus 1 and 2 models. The consensus 2 model with the use of average probability values gave the best AUC value for each validation set (Table 3). The AUC value of the consensus 2 model was 0.84 even for the external validation set based on the average probability values. Moreover the AUC values of the training and the validation sets were not far from each other.

**Table 3:** The AUC values of the prepared consensus models based on the six primary models for training, CV, internal and external validation

Validation	Consensus 1 (MLP + GBT)			Consensus 2 (MLP + GBT)		
	Min	Ave	Max	Min	Ave	Max
Training	0.87	0.88	0.87	0.91	**0.93**	0.91
CV	0.76	0.79	0.76	0.84	**0.86**	0.84
Test	0.77	0.79	0.76	0.83	**0.85**	0.83
External	0.75	0.77	0.74	0.83	**0.84**	0.82

Abbreviations can be found in the beginning of the text. The AUC values of the best model are marked with bold numbers

For the consensus 1 model, the number of molecules was the same as for the primary models. The total number of different molecules in consensus 2 model (MLP + GBT) is more than 23,000 (Table 4).

**Table 4:** The amount of molecules and the ratio of actives in consensus 2 model together with the ratio of excluded molecules compared to the primary models

Samples	Validation	Consensus 1 (MLP + GBT)	Consensus 2 (MLP + GBT)	Active molecules in consensus 2 (%) (MLP + GBT)	Ratio of excluded molecules (consensus 2 for MLP + GBT)
MLP	Training	25,013	14,105	52.2	0.44
	CV	25,013	12,526	49.3	0.50
	Test	10,720	5738	50.7	0.46
	External	9983	4802	23.0	0.52

**Fig. 4:**
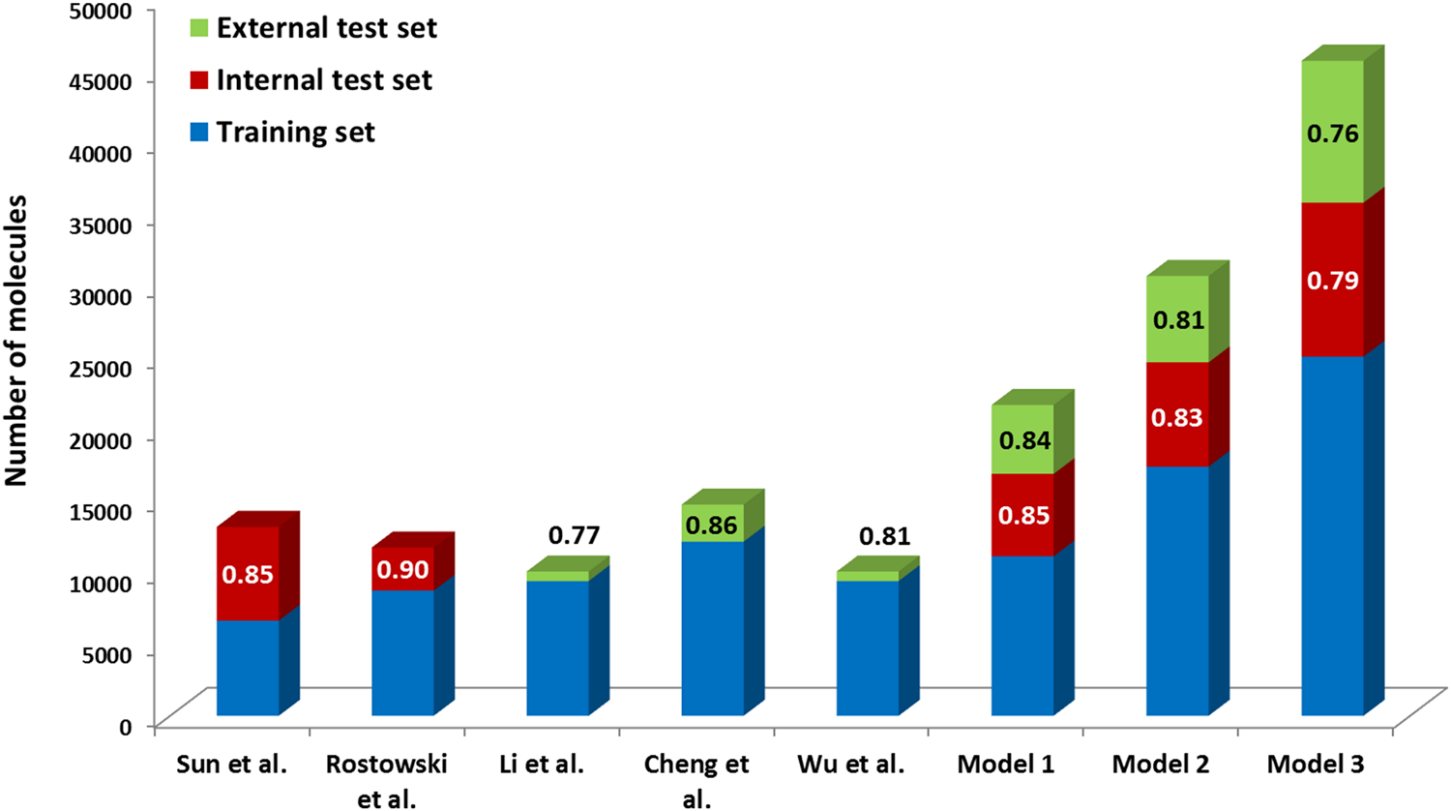
The number of used molecules in the previous studies compared to the top three models in our study. Sun et al. [15], Rostkowski et al. [40], Li et al. [41], Cheng et al. [17], and Wu et al. [18] can be found in the reference list. Model 1: consensus 2 model based on the MLP + GBT algorithms; model 2: consensus 2 model based on the GBT algorithm and model 3: consensus 1 model based on the GBT algorithm. Our selected models can be found in Table 1 and 3 marked with bold

The AUC values of the best three models were compared to that of the previous literature models. Comparison was done with five other studies, where the authors used the AUC values as the performance parameter of their model (Fig. 4). The numbers of used molecules – thus the covered chemical space—was clearly larger than the previous studies in each case, and the AUC values were in the same scale. AUC values are indicated on the diagram for internal and external test sets (previous studies typically applied only one of these).

The AUC, Matthews Correlation Coefficient (MCC), sensitivity (Sn) and Specificity (Sp) values of the top three models are compared with the above mentioned previous studies Tables 5, 6 and 7 (where available).

**Table 5:** The AUC values of the best three models compared to the previous literature models

	AUC values		References
	Internal test set	External test set	
Sun et al.	0.85	–	[15]
Rostkowski et al.	0.90	–	[40]
Li et al.	–	0.77	[41]
Cheng et al.	–	0.86	[17]
Wu et al.	–	0.81	[18]
Model 1	0.85	0.84	–
Model 2	0.83	0.81	–
Model 3	0.79	0.76	–

**Table 6:** Comparison of the Matthews Correlation Coefficients of the top three models and the previous studies

	MCC values		Reference
	Cross-validation	External test set	
Wu et al	–	0.35	[18]
Cheng et al	0.49	–	[17]
Li et al	–	0.32	[41]
Model 1	0.61	0.48	–
Model 2	0.53	0.42	–
Model 3	0.44	0.31	–

Model 1: consensus 2 model based on the MLP + GBT algorithms; model 2: consensus 2 model based on the GBT algorithm and model 3: consensus 1 model based on the GBT algorithm

**Table 7:** Comparison of the sensitivities (Sn) and specificities (Sp) of the top three models and the previous studies

	Cross-validation		External test set		References
	Sn	Sp	Sn	Sp	
Wu	–	–	0.29	0.97	[18]
Cheng et al	0.63	0.85	–	–	[17]
Li et al	–	–	0.32	0.77	[41]
Model 1	0.82	0.79	0.82	0.76	–
Model 2	0.78	0.75	0.79	0.70	–
Model 3	0.73	0.71	0.67	0.71	–

Model 1: consensus 2 model based on the MLP + GBT algorithms; model 2: consensus 2 model based on the GBT algorithm and model 3: consensus 1 model based on the GBT algorithm

While the AUC values are comparable with the previous studies, the MCC values, sensitivities and specificities are slightly or remarkably better. In particular, our models show a greatly improved performance in terms of the balance of sensitivity and specificity.

## Conclusion

Our study provides a large-scale classification model of CYP 2C9 mediated drug interaction potential based on more than 46,000 experimentally tested compounds. The applied algorithms, RPropMLP and GBT were good candidates providing appropriate models for large databases. The use of interaction fingerprints and docking scores gave slightly worse results than the other two primary datasets (typical 1–3D descriptors, molecular and pharmacophore fingerprints), but together, the AUC values of the consensus models have increased. The applied probability threshold determination based on the ROC curves has greatly improved the accuracy of our models in each case. From the consensus modeling point of view, the voting-based consensus 2 models were better than the consensus 1 models, but both gave good results. The AUC values were 0.84 and 0.85 for the external and internal test sets in the case of our best model (consensus 2 with MLP + GBT algorithms). Considering the unprecedented number of diverse molecules used for modeling (and the classification performance being comparable to earlier studies employing much smaller datasets), the resulting classification models are suitable for use in drug discovery workflows.

## Electronic supplementary material

Below is the link to the electronic supplementary material.

Supplementary file1 (DOCX 801 kb)

## Data Availability

All the used primary data are cited properly in the manuscript. The used software and program codes are cited in the manuscript. An additional KNIME workflow can be found in the electronic supplementary material.

## Abbreviations

AUC: Area under the ROC curve
Ave: Average
CYP: Cytochrome P450
DS: Docking score
ECFP: Extended connectivity fingerprint
GBT: Gradient boosted tree
IFP: Interaction fingerprint
Max: Maximum
MD: Molecular descriptors
Min: Minimum
P: Probability
PFP: Pharmacophore fingerprint
ROC: Receiver operating characteristic curve
RPropMLP/MLP: Multi-layer feed-forward of resilient backpropagation network
