# Supplementary material for: Large-scale evaluation of cytochrome P450 2C9 mediated drug interaction potential with machine learning-based consensus modeling
Source: J Comput Aided Mol Des. 2020 Mar 27;34(8):831–9. doi: 10.1007/s10822-020-00308-y (PMC7320947; doi:10.1007/s10822-020-00308-y)

**Supplementary material**

**Large-scale evaluation of Cytochrome P450 2C9 mediated drug interaction potential with machine learning-based consensus modeling**

Anita Rácz^1^, György M. Keserű^2,*^

^1^Plasma Chemistry Research Group, Research Centre for Natural Sciences, Magyar tudósok krt. 2, Budapest 1117, Hungary

^2^Medicinal Chemistry Research Group, Research Centre for Natural Sciences, Hungarian Academy of Sciences, Magyar tudósok krt. 2., Budapest 1117, Hungary

*Correspondence: [keseru.gyorgy@ttk.hu](mailto:keseru.gyorgy@ttk.hu)

**Figure S1A-E.** Comparison of the three Pubchem datasets based on the five druglikeness properties: Molecular weight (MW); logP values; number of nitrogen and oxygen atoms; number of hydrogen bond acceptors (HBA) and donors (HBD). AID777 dataset is marked by green, AID1851 dataset is marked by blue and AID883 dataset is marked by orange in the histograms.


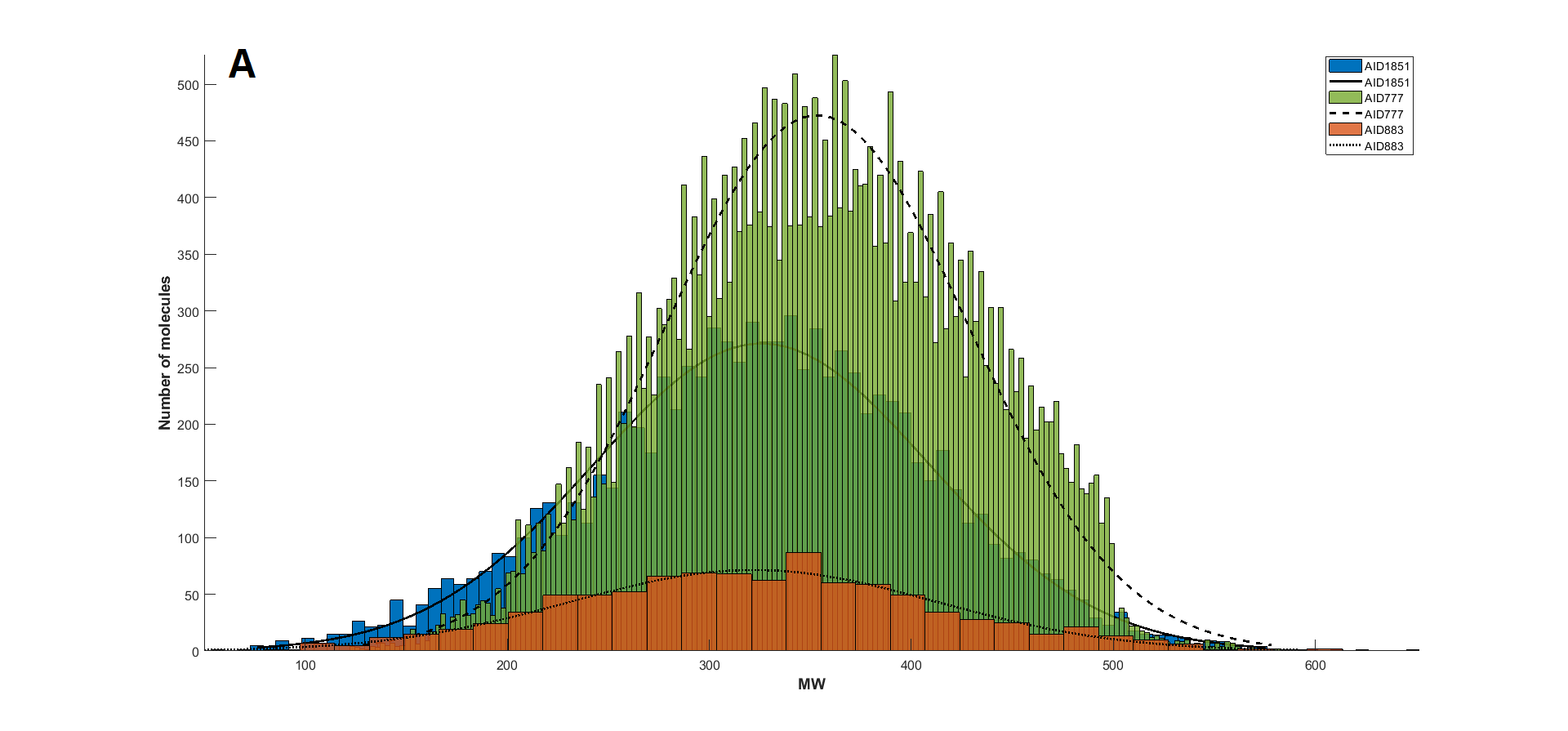


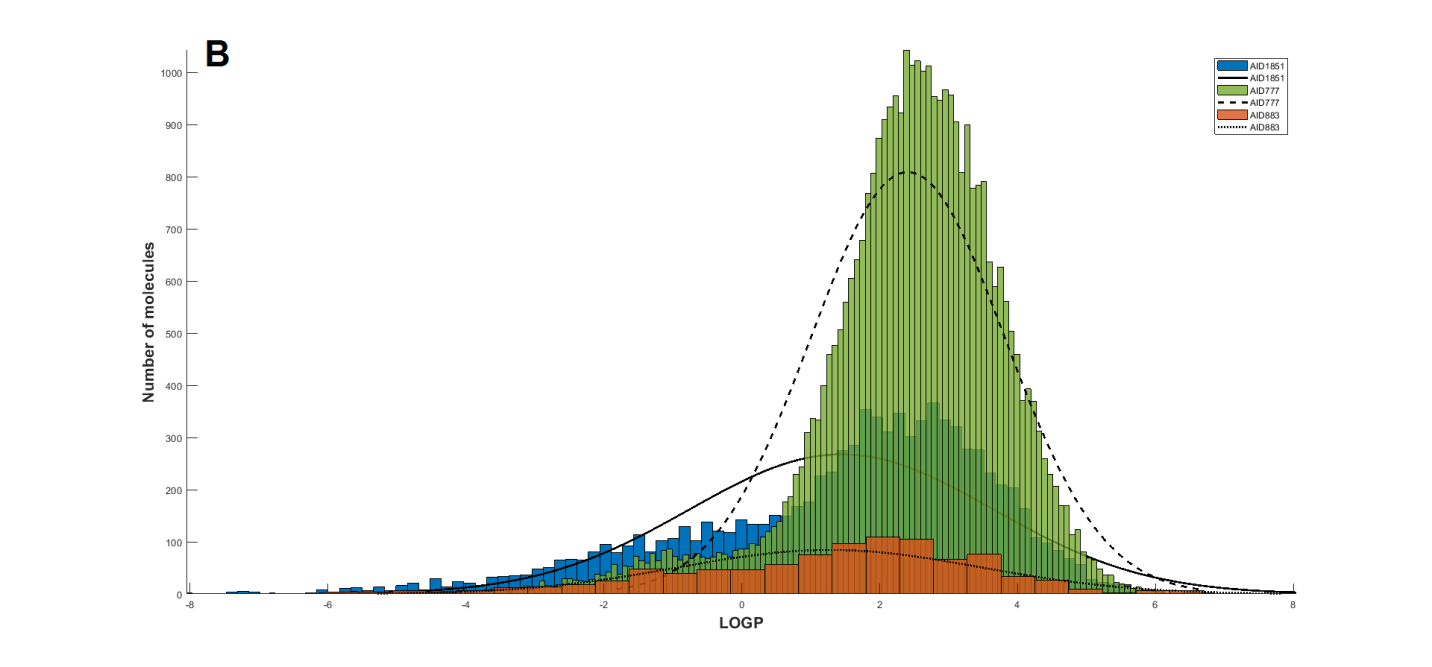


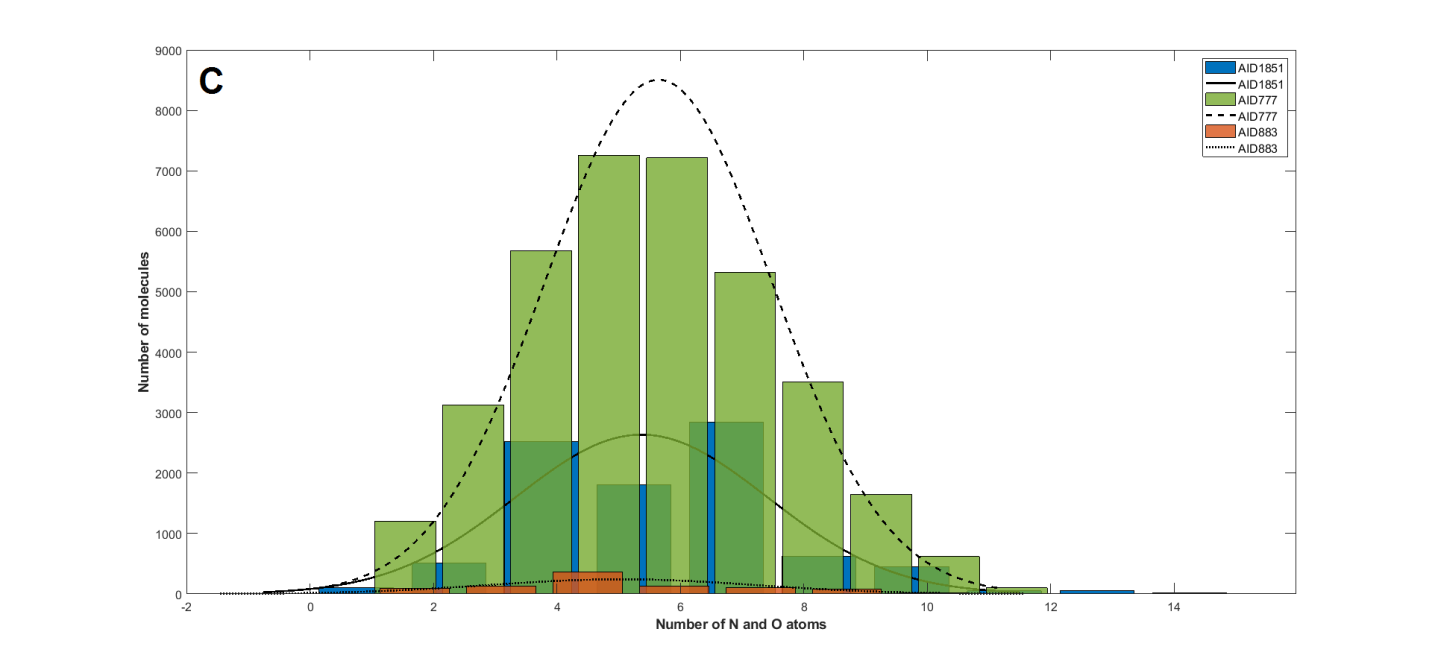


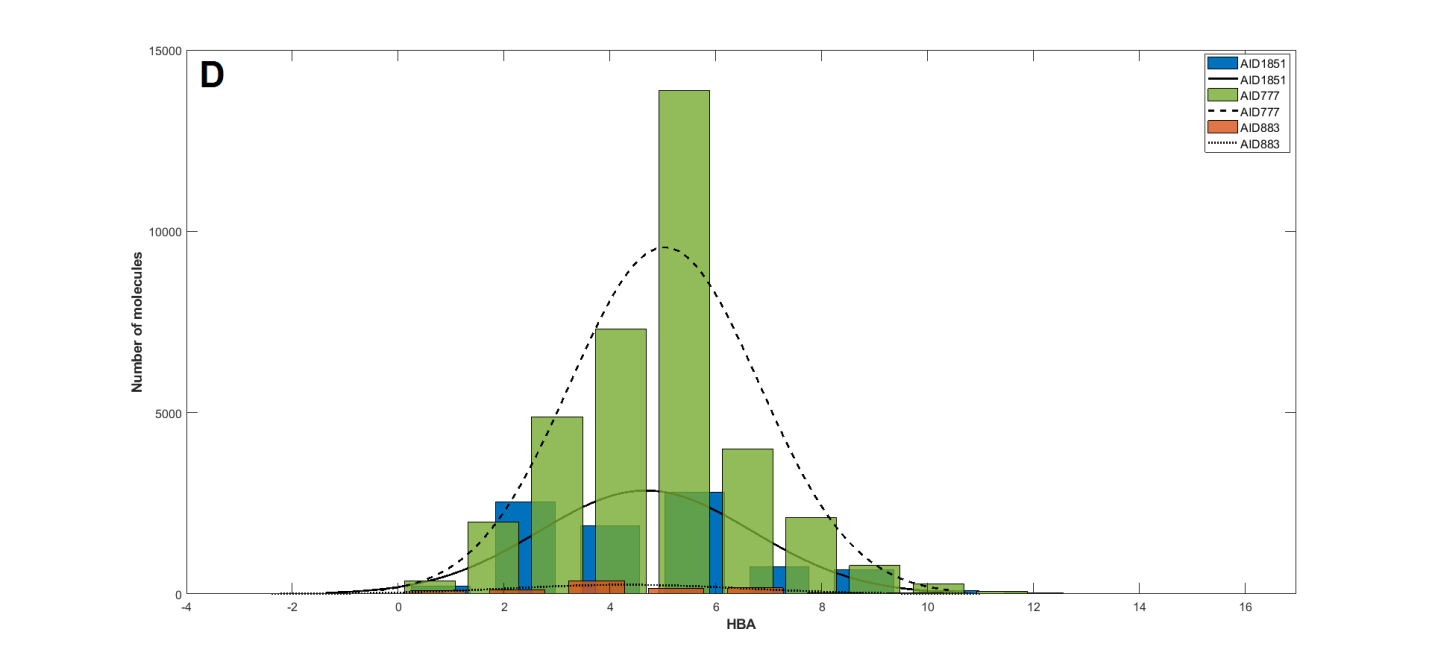


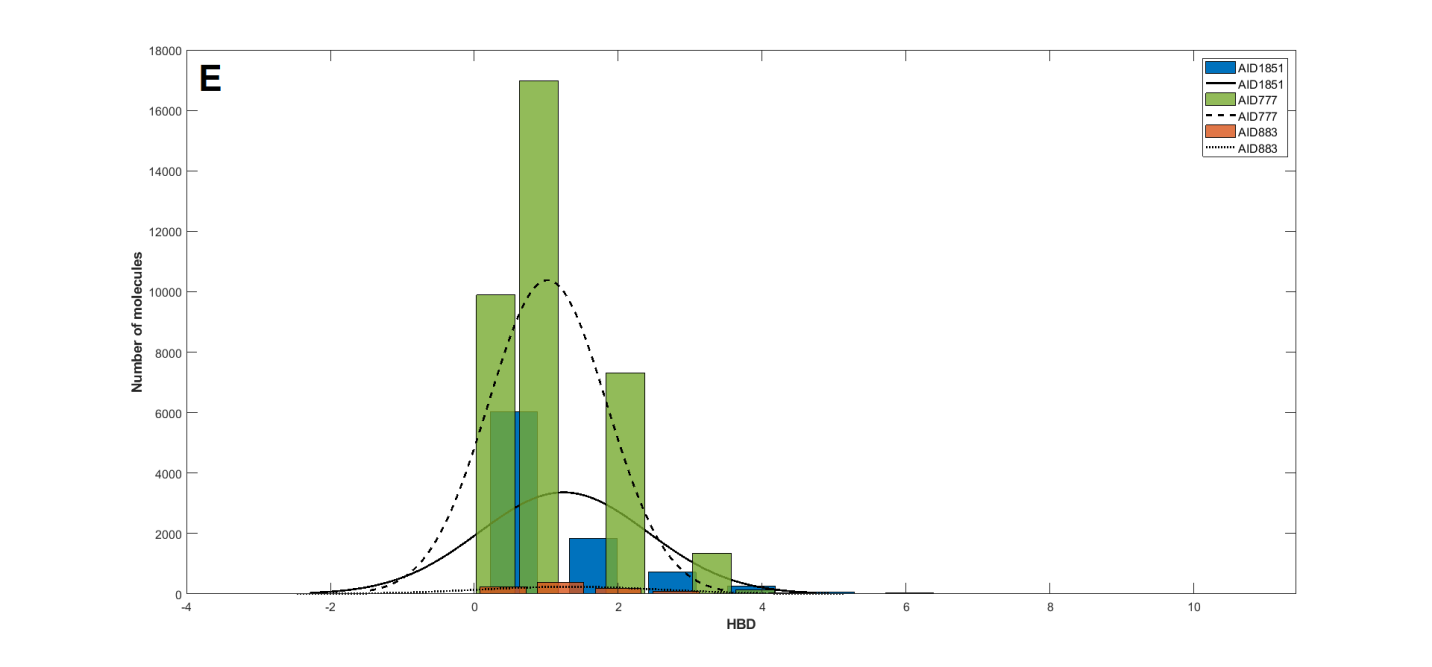

Supplement: Supplementary file 1 — Supplementary file1 (DOCX 801 kb) [file 10822_2020_308_MOESM1_ESM.docx]
